# Differentiating people who use cannabis heavily through latent class analysis

**DOI:** 10.1186/s13011-023-00540-3

**Published:** 2023-06-01

**Authors:** Arturo Alvarez-Roldan, Teresa García-Muñoz, Juan F. Gamella, Iván Parra, Maria J. Duaso

**Affiliations:** 1 Department of Social Anthropology, University of Granada, 18071 Granada, Spain; 2 Department of Quantitative Methods, University of Granada, Granada, Spain; 3 Florence Nightingale Faculty of Nursing, Midwifery and Palliative Care, King’s College London, London, UK

**Keywords:** Cannabis, Marijuana, Heavy users, Latent class analysis, Patterns of use, Cannabis clubs

## Abstract

**Background:**

People who use cannabis daily or near-daily vary considerably in their daily dosage and use frequency, impacting both experienced effects and adverse consequences. This study identified heavy cannabis user groups according to consumption patterns and factors associated with class membership.

**Methods:**

We conducted a cross-sectional study of 380 Spanish residents (61.8% male; average age = 30.3 years) who had used cannabis ≥ 3 days/week throughout the past year. Participants were recruited through chain referral and cannabis social clubs. We applied latent class analysis (LCA) to cluster participants according to use intensity. LCA indicators included frequency of weekly cannabis use, joints smoked each day, cannabis dosage, and if cannabis was consumed throughout the day or only at specific times. Associations between class membership and socio-demographics, use patterns, motives, supply sources, adverse outcomes, and use of other substances were measured using ANOVA and chi-squared tests. Multinomial regression identified the factors associated with latent class membership.

**Results:**

Three latent classes (moderately heavy: 21.8%, heavy: 68.2%, very heavy: 10%) had average weekly cannabis intakes of 2.4, 5.5, and 18.3 g, respectively. Very heavy users were older (${\chi }^{2}$=17.77, *p* < 0.01), less educated ${(\chi }^{2}$=36.80, *p* < 0.001), and had used cannabis for longer (*F* = 4.62, *p* = 0.01). CAST scores (*F* = 26.51, *p* < 0.001) increased across the classes. The prevalence of past-month alcohol use was lower among the heaviest users (${\chi }^{2}$=5.95, *p* = 0.05). Cannabis was usually obtained from a club by very heavy users (${\chi }^{2}$=20.95, *p* < 0.001).

**Conclusions:**

People who use cannabis heavily present three groups according to frequency and quantity of cannabis consumption. Use intensity is associated with increased cannabis-related problems. Differences among heavy users must be considered in harm reduction interventions in cannabis clubs and indicated prevention.

## Background

Cannabis is the most commonly used illicit drug worldwide [1], and Spain has one of the highest consumption rates in Europe [2]. In 2019, 10.5% of the population (aged 15–64 years) had used cannabis in the past year. In total, 8% had used cannabis in the past 30 days, and 2.9% used it daily [3].

Research has found that daily or near-daily (DND) users (i.e., ≥ 20 days of consumption during the previous month) account for almost 80% of total cannabis consumption [4–6]. Their use patterns require more detailed analysis because they consume larger doses and take it several times during the waking day [7–10]. Therefore, they are more vulnerable to acute and chronic health or psychological harms associated with cannabis use, including impaired psychomotor and cognitive functioning, memory deficits, dependence, respiratory impairments (including bronchitis), onset or amplification of psychosis in predisposed individuals, and driving impairment with risk of traffic injuries [11–15]. Daily use in adolescents and young adults is also associated with early school leaving, increased risk of using other illicit drugs, and cognitive and psychological deterioration [12–15].

Experts agree that standardized tools for measuring cannabis exposure are necessary to unify the evidence on the antecedents, correlates, and consequences of use [16]. Surveys tend to distinguish people who use cannabis (PWUC) heavily based on frequency of use, and rarely ask about average daily intake. However, DND users consume remarkably diverse amounts of cannabis. For instance, Gamella and Jiménez [17] found that the number of joints smoked monthly by a sample of 81 Spanish daily long-term cannabis users ranged from 10 to 300; similar findings were reported internationally [10, 18]. Therefore, it seems that a more accurate assessment of cannabis use requires assessing both, frequency and quantity. Indeed, Tomko et al. [19] have shown that the sum of the quantity of cannabis used (average grams per administration _*_ number of joints/day) and the frequency of use in the past 30 days significantly improves the prediction of urine cannabinoid level and cannabis-related problems.

This study aimed to analyze groups of heavy users according to their use patterns, and whether their consumption and its consequences varied. We hypothesized that the “heavy use” category comprises a variety of use patterns associated with different vulnerability for cannabis-related problems. We chose latent class analysis (LCA)—a model-based clustering method—to uncover these groups in a sample of heavy users. This method allows to identify different groups, accurately characterizes membership, and predicts which case is likely to belong to each group.

Previous studies have utilized LCA to identify cannabis user types according to several sets of variables. Craft et al. [20] identified seven classes of PWUC characterized by the probability of using different products. Herbal cannabis, sinsemilla, and hashish were associated with increased dependence, whereas the consumption of concentrates was associated with diagnosis of mental health disorders. Davis et al. [21] distinguished four groups in a sample of twins and siblings based on the concurrent and simultaneous use of cannabis with tobacco, alcohol, and other illicit drugs. Simultaneous use of cannabis and tobacco was associated with the most problematic outcomes, including depression, illicit drug use, and cannabis use disorders. Two studies found four and five subgroups, respectively, of persons who use cannabis based on the products they used and their past-month use frequency/intensity [22, 23]. Heaviest users, who consumed plant products, concentrates, or both frequently and spent more time high, were more susceptible to experiment adverse consequences. Another two studies uncovered four and five classes of users, respectively, according to consumption frequency, quantity/intensity, and cannabis-related problems [24, 25]. Both studies identified three groups among heavy users with increasingly more negative consequences.

To conduct our LCA, we included only variables affecting the magnitude of exposure to cannabis and its effects. We excluded consumption method because all our participants principally smoked cannabis in joints. Smoking combusted cannabis is the predominant method among PWUC in Spain. In 2019, 98% of past-month cannabis users reported smoking it in a joint; 87% mixed it with tobacco [3]. We chose for the post-LCA analysis variables describing social, health, or behavioral characteristics or outcomes of cannabis use relevant to public health. We followed the previous literature to select these indicators: sociodemographic factors, settings and sources of cannabis use, other drug use, and health outcomes [26].

This study aims to contribute to the knowledge of PWUC heavily in Spain, their demographics, use patterns, and experienced consequences. We applied LCA to: [1] identify classes in heavy users according to their cannabis exposure magnitude; [2] examine if there were associations between class membership and other variables (socio-demographics, characteristics of cannabis use, motives, sources of supply, adverse outcomes, and concurrent use of other substances); and [3] identify which of the examined variables remained as predictors of class membership in a multinomial regression model.

## Methods

### Participants and procedure

We recruited 380 individuals who had consumed cannabis ≥ 3 days/week in the past 12 months (average cannabis use days/week = 6.3; 61.8% male; average age = 30.3; age range = 18–76). The Dutch Cannabis Dependence (CanDep) study employed the same eligibility criterion [27]. Participants were recruited through chain referral and cannabis social clubs in Spain from January 2017 to May 2019. Involvement was voluntary and anonymous. Participants were informed about the research aims, the institutions responsible and the funding body. They provided consent to respond to a paper-and-pencil self-administered questionnaire (average completion time: 40 min). In total, 142 participants (37%) were members of cannabis clubs. These clubs are registered non-profit associations that organize the production and distribution of cannabis among their adult members. We invited members of some cannabis clubs to participate with the help of some of the clubs’ staff. They sent us back the completed questionnaires. Other club members appeared in the networks of users found by snowball sampling. Various collaborators help us to follow these networks and to contact participants in different settings.

### Variables

We selected four variables describing the intensity of cannabis use for LCA:

- The *number of weekly cannabis use-days*.
- The *number of joints typically smoked in a use-day* was estimated by responding to the following questions: “How many joints did you smoke the last time you smoked alone?” “How many joints did you smoke the last time you smoked with others?” “With how many individuals did you share each joint?” For example, smoking one joint alone and three with another individual gives a total of 2.5 joints. If the last consumption day was atypical, participants responded to the same questions regarding a regular cannabis use day.
- Cannabis dosage was estimated by self-report of *the number of joints made with one gram of cannabis*. This method has been previously validated to better estimate average doses per joint at group level compared to other methods such as using a prompt card with real-size pictures of a ruler and different dosages [28]. The estimated median in our sample was 0.28 g per joint, very near the 0.25-gram “standard unit joint” [28].
- *All-day consumption*. Finally, individuals had to report if they usually consumed cannabis throughout the waking day or only at specific times (morning, afternoon, evening, before going to bed).

The variables selected for the post-LCA analysis were:

- *Socio-demographics.* Age (by cohorts; ordinal variable), sex (male dummy variable), marital status (married or with a partner versus other; categorical variable), education (primary, secondary, vocational training, baccalaureate, or university; ordinal variable), employment (employed, unemployed, or inactive; categorical variable), and monthly income (< 301€, 301–900€, > 901€; ordinal variable).
- *Characteristics of cannabis use.* Number of years of cannabis use (continuous). Methods of cannabis consumption in the last month (smoked mixed with tobacco, smoked without tobacco, vaporized, eaten, or drunk; all yes or no). Cannabis use settings in the past month (at home, outdoors [street, square, park, countryside, beach], in a club, in a vehicle; all yes or no).
- *Motives for use*. We used the Spanish version of the 25-item Marijuana Motives Measure (MMM) questionnaire [29–32]. Participants rated each item on a 5-point Likert scale from 1 (*almost never/never*) to 5 (*almost always/always*).
- S*ource of supply.* Purchased in the illegal market, obtained in a cannabis club, home-grown, obtained for free (all yes or no).
- *Adverse consequences.* Experienced cannabis-related problems in the last year (health, psychological, at school or university, family, financial; all yes or no); had received a fine because of cannabis use in the past year (yes or no). Cannabis use disorder (CUD) and cannabis dependence were assessed using the full Spanish version of the Cannabis Abuse Screening Test (CAST) [33–35] which assesses the frequency of the following events in the past 12 months: “Have you smoked cannabis before mid-day?”; “Have you smoked cannabis when you were alone?”; “Have you had memory problems when you smoked cannabis?”; “Have friends or family members told you that you should reduce or stop your cannabis use?”; “Have you tried to reduce or stop your cannabis use without succeeding?”; “Have you had problems because of your cannabis use (argument, fight, accident, poor results at school, etc.)?”. All items are answered on a five-point scale from 0 (never) to 4 (very often). Total scores range from 0 to 24. The cut-off points used, according to DSM-IV criteria, were: 9 for CUD and 12 for cannabis dependence (Cronbach’s alpha: 0.75, sensitivities: 0.74 and 0.57, specificities: 0.69 and 0.84) [36].
- *Use of other substances*: use of tobacco and alcohol in the last month (both yes or no); use of inhalants, cocaine, amphetamine, ecstasy, LSD, sedative-hypnotics, synthetic cannabinoids, and mushrooms in the past 12 months (all yes or no). A dummy variable was created to classify individuals that had taken one or more non-cannabis illicit substances in the past year.

### Statistical analysis

We applied LCA to cluster participants according to the intensity of their cannabis consumption. LCA is a statistical procedure used to identify latent (unobserved) groups within a sample by sharing some characteristics. The latent groups are inferred from patterns of the observed variables used in the modelling. We conducted different LCAs to identify classes of PWUC heavily based on patterns of cannabis use. We selected four variables to develop the LCA: number of weekly cannabis use-days, number of joints smoked on a typical use-day, number of joints made from one gram of cannabis, and cannabis use throughout the waking day. Then, we explored several solutions, beginning with the most parsimonious (i.e., one class) and increasing the number of latent classes by one to determine the model that featured the best data fit.

For reasons of interpretability and parsimony, we did not use models where the smallest class proportion was < 5.2% [37]. The first model with this characteristic was the 4-class model. To determine the number of classes that have the best fit, we considered the Akaike information criterion (AIC), consistent AIC (cAIC), Bayesian information criterion (BIC), sample-size adjusted BIC (aBIC), approximate weight of evidence criterion (AWE). Lower values indicate better fit and parsimony [38]. The Lo–Mendell–Rubin (LMR) test was also computed to compare k-1 class and k class models: higher p values suggest that the k class model does not fit the data significantly better than a model with one less class. Finally, we estimated the entropy (ranging from 0 to 1) where values > 0.80 indicate a successful classification of individuals into classes.

After deciding the number of latent classes and using the latent class posterior probabilities, individuals were classified in their most likely class. In the next step, ANOVA (Scheffe as post-hoc test) and chi-squared (and Fisher’s exact for groups with small sample size) tests were used to compare continuous and categorical variables, respectively. In the case of multiple comparisons, Holm-Bonferroni correction was performed. Finally, we applied multinomial regression to identify the factors associated with latent class membership; this regression only included statistically significant variables from the previous step. All analyses were conducted in STATA (version 15).

## Results

### Latent class model

Comparisons of the LCA model-fit statistics suggested that the three-class model provided the best fit (Table 1).

**Table 1:** Fit indices for one- to three-class models

Number of classes	AIC	cAIC	BIC	aBIC	AWE	Entropy	LMR statistic (p-value)	Proportion in smallest class
1	4968.70	4996.30	4996.28	4974.07	5057.86	--	--	--
2	4836.35	4883.66	4883.63	4883.63	4990.91	0.88	117.92 (p < 0.001)	13.41%
3	4504.68	4571.71	4571.66	4517.73	4723.65	0.93	283.05 (p < 0.001)	10.71%

AIC: Akaike Information Criterion; cAIC: consistent AIC; BIC: Bayesian Information Criterion: aBIC: sample size adjusted BIC; AWE: Approximate Weight of Evidence criterion. LMR: Lo–Mendell–Rubin test

**Fig. 1:**
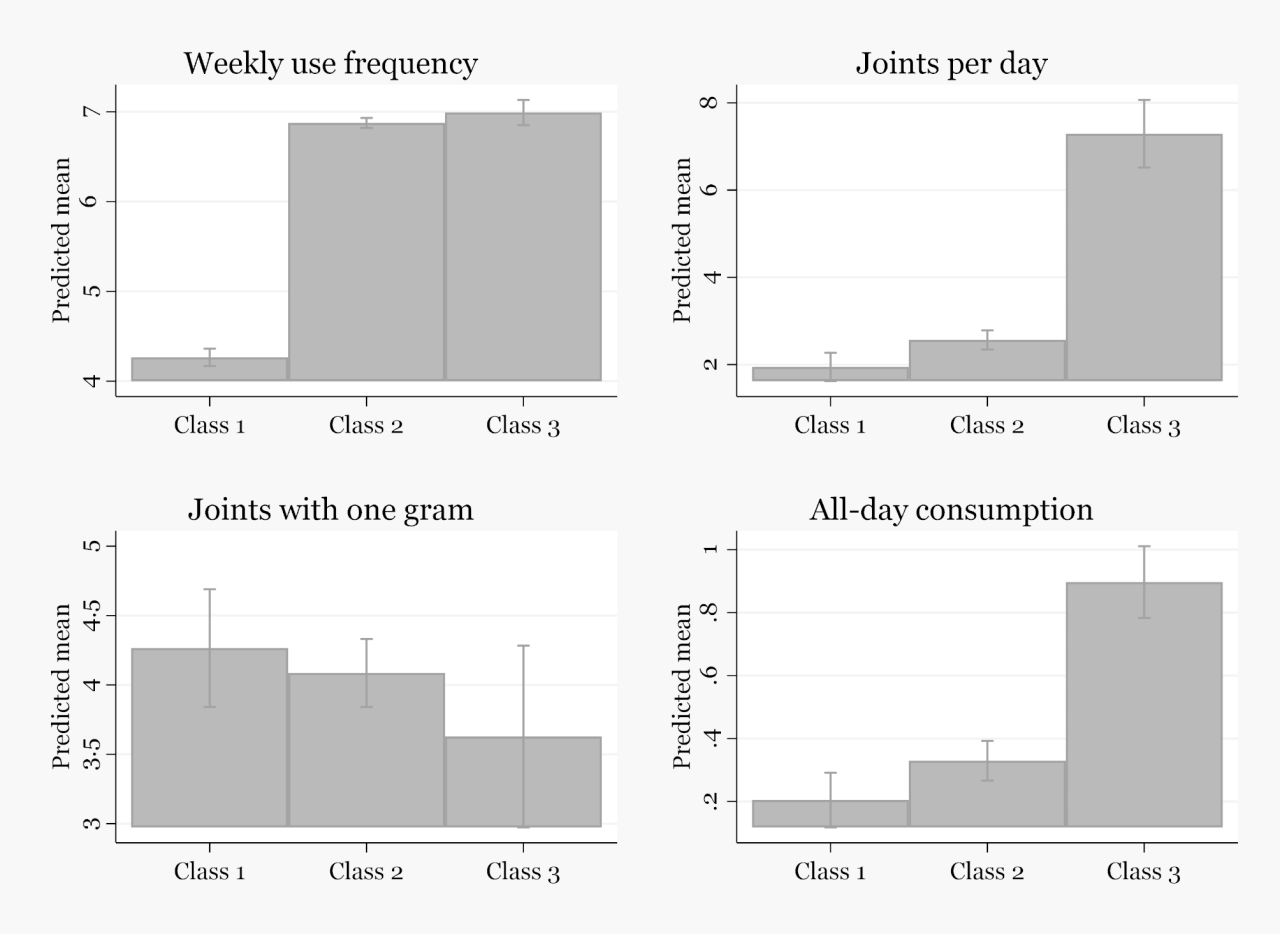
Predicted mean of variables in LCA analysis by classes

Figure 1 presents the predicted mean of all variables used in the LCA analysis by class. The three latent classes show a clear division according to cannabis use intensity.

- *Class 1* comprised 21.8% of the sample and had the following characteristics. Usage days per week: M = 4.27, SD = 0.77; joints smoked per consumption day: M = 1.9, SD = 1.6; dose of cannabis per joint: M = 264 mg, SD = 94; weekly consumption: M = 2.24 g, SD = 2.02; 20.5% consumed cannabis throughout the waking day. This subgroup was labeled *moderately heavy*.
- *Class 2* comprised 68.2% of the sample and had the following characteristics. Usage days per week: M = 6.88, SD = 0.33; joints smoked per consumption day: M = 2.6, SD = 1.3; dose of cannabis per joint: M: 305 mg, SD = 154; weekly consumption: M = 5.49 g, SD = 4.68; 33.6% used cannabis throughout the waking day. This subgroup was labeled *heavy*.
- *Class 3* consisted of 10% of the sample; they were all daily users. Their other characteristics were as follows. Joints smoked per consumption day: M = 7.6, SD = 1.9; dose of cannabis per joint: M = 344 mg, SD = 161; weekly consumption: M = 18.25 g, SD = 8; 89.5% took cannabis throughout the waking day. This subgroup was labeled *very heavy*.

### Bivariate associations

#### Socio-demographics

Differences among classes for socio-demographic variables are presented in Table 2. There were significant intergroup differences regarding age—from youngest to oldest: moderately heavy, heavy, and very heavy. Education levels were similar between classes 1 and 2. However, the percentage of respondents with education below university level was significantly higher in the very heavy group. Similarly, unemployment was significantly higher in the very heavy group, and there were more inactive people (mainly students) among the moderately heavy users. The proportions of being employed and monthly income were similar between the three classes. There was no difference in the proportion of being married or having a partner.

**Table 2:** Latent class membership by socio-demographics

	N(%)				Significance
	Overall 380(100.0%)	Class 1: moderately heavy 83(21.8%)	Class 2: heavy 259(68.2%)	Class 3: very heavy 38 (10%)	
Age					${\chi }^{2}$=17.77 p=0.007
18–20 years old	36(9.5)	15(18.1)	15(5.8)	6(15.8)	
21–30 years old	199(52.4)	43(51.2)	143(55.2)	13(34.2)	
31–40 years old	94(24.7)	15(18.0)	67(25.9)	12(31.6)	
> 40 years old	51(13.4)	10(12.1)	34(13.3)	7(18.4)	
Male	235(61.8)	53(63.9)	152(58.6)	30(78.9)	${\chi }^{2}$=5.94 p=0.051
Married/with partner	89(23.4)	14(16.9)	65(25.1)	10(26.3)	${\chi }^{2}$=2.57 p=0.277
Education					${\chi }^{2}$=36.80 p<0.0001
Primary	19(5.0)	2(2.4)	11(4.3)	6(15.8)	
Secondary	46(12.2)	6(7.23)	29(11.3)	11(29.0)	
Vocational Training	40(10.6)	10(12.0)	22(8.6)	8(21.0)	
Baccalaureate	45(11.9)	9(10.8)	31(12.1)	5(13.2)	
University	227(60.2)	56(67.5)	163(63.7)	8(21.0)	
Employment					${\chi }^{2}$=10.06 p=0.039
Employed	237(62.4)	49(59.0)	166(64.1)	22(57.9)	
Unemployed	52(13.7)	11(13.2)	30(11.6)	11(28.9)	
Inactive	91(23.9)	23(27.7)	63(24.3)	5(13.2)	
Monthly income					${\chi }^{2}$=1.44 p=0.837
Less 301 €	75(22.3)	17(24.3)	49(21.0)	9(27.3)	
301–900 €	115(34.2)	25(35.7)	81(34.8)	9(27.3)	
Higher 901 €	146(43.4)	28(40.0)	103(44.2)	15(45.4)	

#### Characteristics of cannabis use

Table 3 panel A shows intergroup differences regarding cannabis use characteristics. The mean number of years between first and current use was 14.2 (SD = 9.1). Class 3 reported the lengthiest period of use (range: 12.2 years [class 1] to 17.5 years [class 3]). However, there was no significant difference regarding the average age of cannabis use onset: 16.1 years (SD = 3.9) overall. The most typical form of cannabis use in the past month was smoking mixed with tobacco in all classes with significant differences (range: 86.7% [class 1] to 100% [class 3]). There were no significant intergroup differences regarding other modes of cannabis administration in the past 30 days: smoked without tobacco, vaped, eaten, or drunk. In the past month, 91.8% had used cannabis at home, 42.1% outdoors (street, square, park, countryside, beach), 39.7% in a cannabis social club, and 31.6% inside a vehicle. Intergroup differences regarding all cannabis use settings in the past month were significant. Class 1 reported the lowest proportion of current use in all the locations, and class 3 the highest, except at home where class 2 was slightly more prevalent. The prevalence of individuals who had consumed cannabis in a club in the last month increased through the classes (range: 19.3% [class 1] to 63.2% [class 3]). Almost half of class 3 had used cannabis in an automobile in the past month. The proportion decreased to 21.7% in class 1

**Table 3:** Latent class membership by several characteristics

	Mean (SD)/N (%)				Significance	
	Overall 380(100.0%)	Class 1: moderately heavy 83(21.8%)	Class 2: heavy 259(68.2%)	Class 3: very heavy 38 (10%)		
(A) Latent class membership by characteristics of cannabis use						
Nº of years used cannabis	14.2 (9.1)	12.2 (9.1)	14.4 (8.9)	17.5 (9.4)	F = 4.62 p = 0.010	
Age of onset	16.1(3.9)	16.3(3.7)	16.1(4.0)	15.2(3.1)	F = 4.59 p = 0.101	
Mode of administration						
Smoked mixed with tobacco	351(92.4)	72(86.7)	241(93.0)	38(100.0)	${\chi }^{2}$=7.03 p=0.030	
Smoked without tobacco	118(31.0)	29(34.9)	79(30.5)	10(26.3)	${\chi }^{2}$=1.02 p=0.600	
Vaporized	63 (16.6)	12(14.5)	42(16.2)	9(23.7)	${\chi }^{2}$=1.68 p=0.431	
Eaten or drunk	67(17.6)	12(14.5)	46(17.8)	9(23.7)	${\chi }^{2}$=1.54 p=0.464	
Setting						
Home	349(91.8)	68(81.9)	246(95.0)	35(92.1)	${\chi }^{2}$=14.30 p=0.001	
Outdoors	160(42.1)	24(28.9)	118(45.6)	18(47.4)	${\chi }^{2}$=7.62 p=0.022	
Club	151(39.7)	16(19.3)	111(42.9)	24(63.2)	${\chi }^{2}$=24.27 p<0.0001	
Vehicle	120(31.6)	18(21.7)	85(32.8)	17(44.7)	${\chi }^{2}$=6.99 p=0.030	
(B) Latent class membership by reasons for use						
Social	2.4(0.9)	2.4(0.9)	2.3(0.9)	2.4(1.0)	F = 0.33 p = 0.725	
Expansion	2.4(1.0)	2.2(1.0)	2.5(1.0)	2.2(1.0)	F = 3.07 p = 0.047	
Coping	2.7(0.9)	2.4(0.9)	2.7(0.9)	2.9(1.0)	F = 3.74 p = 0.024	
Conformity	1.3(0.4)	1.3(0.3)	1.3(0.5)	1.2(0.2)	F = 1.26 p = 0.285	
Enhancement	3.8(0.7)	3.8(0.8)	3.7(0.7)	3.9(0.7)	F = 0.77 p = 0.464	
Medical	29(7.6)	8(9.6)	18(7.0)	3(7.9)	${\chi }^{2}$=0.65 p=0.723	
(C) Latent class membership by sources of supply						
Purchased	284(74.7)	64(77.1)	193(74.5)	27(71.0)	${\chi }^{2}$=0.53 p=0.759	
Obtained for free	90(23.7)	28(33.7)	57(22.0)	5(13.2)	${\chi }^{2}$=7.37 p=0.025	
Club	142(37.4)	16(19.3)	103(39.8)	23(60.5)	${\chi }^{2}$=20.95 p<0.0001	
Home grown	34(9.0)	2(2.4)	30(11.6)	2(5.3)	${\chi }^{2}$=7.19 p=0.027	

#### Motives for use

Factor analysis of the five potential motives for cannabis use (MMM) suggested enhancement motives predominated for all classes (M = 3.8, SD 0.7), followed by coping (M = 2.7, SD = 0.9), expansion (M = 2.4, SD = 1.0), and social motives (M = 2.4, SD = 0.9). Conformity motives were rare (M = 1.3, SD = 0.4). Several significant intergroup differences emerged regarding motives for cannabis use. Coping motives significantly increased through the classes (range: 2.4 [class 1] to 2.9 [class 3], and class 2 showed higher expansion motives than the other two classes. (Table 3 panel B).

#### Supply sources

Table 3 panel C presents the distribution of supply sources among groups. Typically, cannabis was purchased in all classes. Obtaining cannabis for free (or from sharing with others) was more common in class 1. There were substantial significant intergroup differences in the proportion of users who had obtained cannabis from a social club (range: 19.3% [class 1] to 60.5% [class 3]). Only 9% of respondents had grown their cannabis plants. Class 2 included 11.6% cultivators, in contrast with only 2.4% and 5.3% in classes 1 and 3, respectively.

#### Adverse outcomes

Table 4 panel A provides the bivariate associations between the three subgroups and self-reported adverse outcomes in the past year. Psychological and social troubles were more prevalent than physical problems across subgroups. Intergroup differences emerged concerning the proportion of individuals who had experienced psychological disorders, family disputes, financial problems, or received a fine (higher among very heavy users). There were considerable significant intergroup differences regarding CAST scores. Overall, 59.2% of users reported CUD (CAST ≥ 9) and 36.6% dependence (CAST ≥ 12). The prevalence of CUD (range: 43.4% [class 1] to 89.5% [class 3]) and dependence (range: 21.7% [class 1] to 68.4% [class 3]) increased significantly through the classes.

**Table 4:** Latent class membership by problems and concurrent use of other substances

		N (%)				Significance
		Overall 380(100.0%)	Class 1: moderately heavy 83(21.8%)	Class 2: heavy 259(68.2%)	Class 3: very heavy 38 (10%)	
(A) Latent class membership by problems						
Problems last year						
Health		39(10.3)	8(9.6)	25(9.6)	6(15.8)	${\chi }^{2}$=1.40 p=0.497
Psychological		108(28.4)	16(19.3)	75(29.0)	17(44.7)	${\chi }^{2}$=8.42 p=0.015
At school/university		72(18.9)	14(16.9)	49(18.9)	9(23.7)	${\chi }^{2}$=0.78 p=0.674
Family		73(19.2)	15(18.1)	42(16.2)	16(42.1)	${\chi }^{2}$=14.40 p=0.001
Financial		116(30.5)	25(30.1)	73(28.1)	18(47.4)	${\chi }^{2}$=5.76 p=0.056
Fines		118(31.1)	20(24.1)	80(30.9)	18(47.4)	${\chi }^{2}$=6.60 p=0.037
Cannabis Abuse Screening Test	10.0(4.4)		8.0(3.5)	10.0(4.2)	13.9(5.1)	F = 26.51 p < 0.0001
CUD (CAST ≥ 9)		225(59.2)	36(43.4)	155(60.0)	34(89.5)	${\chi }^{2}$=23.99 p<0.0001
Dependence (CAST ≥ 12)		139(36.6)	18(21.7)	95(36.7)	26(68.4)	${\chi }^{2}$=24.54 p<0.0001
(B) Latent class membership by concurrent use of other substances						
Tobacco (last month)		271(71.3)	52(62.7)	186(71.8)	33(86.8)	${\chi }^{2}$=7.56 p=0.023
Alcohol (last month)		327(86.5)	72(87.8)	227(87.9)	28(73.7)	${\chi }^{2}$=5.95 p=0.051
Inhalants (last year)		38(10.0)	2(2.4)	31(12.0)	5(13.2)	${\chi }^{2}$=6.73 p=0.035
Cocaine (last year)		137(36.1)	19(23.2)	98(37.8)	20(52.6)	${\chi }^{2}$=10.78 p=0.005
Amphetamine (last year)		81(21.4)	10(12.2)	60(23.2)	11(28.9)	${\chi }^{2}$=5.90 p=0.052
Ecstasy (last year)		95(25.1)	19(23.2)	64(24.7)	12(31.6)	${\chi }^{2}$=1.03 p=0.597
LSD (last year)		37(9.8)	3(3.7)	27(10.4)	7(18.4)	${\chi }^{2}$=6.83 p=0.033
Sedative-hypnotics (last year)		70(18.5)	17(20.7)	49(18.9)	4(10.5)	${\chi }^{2}$=1.90 p=0.386
Synthetic cannabinoids (last year)		14(3.7)	1(1.2)	9(3.5)	4(10.5)	${\chi }^{2}$=6.43 p=0.040
Mushrooms (last year)		48(12.7)	8(9.8)	37(14.3)	3(7.8)	${\chi }^{2}$=2.02 p=0.363
Use of ≥ 1 non-cannabis illicit drug (past year)		209(55.1)	37(45.1)	149(57.5)	23(60.5)	${\chi }^{2}$=4.37 p=0.112

#### Use of other substances

Table 4 panel B shows intergroup differences regarding the concurrent use of other substances. Most respondents had smoked tobacco separately in the past month with significant intergroup differences (range: 62.7% [class 1] to 86.8% [class 3]). The percentage of individuals who had drunk alcohol in the last month was significantly lower in the very heavy group than in the others (range: 73.7% [class 3] to 87.9% [class 2]). More than half had used at least one non-cannabis illicit drug in the past 12 months, most commonly cocaine (range: 23.2% [class 1] to 52.6% [class 3]). Significant intergroup differences emerged regarding the consumption of inhalants, amphetamine, LDS, and synthetic cannabinoids in the last year (higher among very heavy users).

### Multivariate associations

We conducted a multinomial regression model with all the variables that were statistically significant in the previous bivariate analyses (Table 5). Almost all the individuals who had consumed cannabis in a club in the past month had also acquired it there. Therefore, we excluded the variable “club as use setting” in the regression model and kept only “club as a supply source.“ In adverse outcomes, we included CAST scores instead of the cut-off points for CUD or dependence. Table 5 shows the resulting model.

**Table 5:** Multinomial logistic regression

	Moderately heavy (as ref.) vs. heavy		Moderately heavy (as ref.) vs. Very heavy		Heavy (as ref.) vs. Very heavy	
	Coefficient	95% Conf. interval	Coefficient	95% Conf. interval	Coefficient	95% Conf. interval
Socio-demographics						
*Age*						
18–20 years old	Ref.	-	Ref.	-	Ref.	-
21–30 years old	1.413*	(0.214, 2.611)	-0.364	(-2.748, 2.020)	-1.777	(-3.949, 0.395)
31–40 years old	0.895	(-0.774, 2.564)	-1.795	(-4.928, 1.339)	-2.689	(-5.463, 0.084)
> 40 years old	0.532	(-1.712, 2.776)	-3.232	(-7.642, 1.178)	-3.764	(-7.705, 0.177)
*Male*	-0.324	(-1.005, 0.358)	-0.286	(-1.610, 0.038)	0.037	(-1.128, 1.203)
*Education*						
Primary	Ref.	-	Ref.	-	Ref.	-
Secondary	-0.578	(-2.759, 1.602)	-1.843	(-4.456, 0.769)	-1.265	(-2.967, 0.437)
Vocational Training	-1.275	(-3.400, 0.850)	-2.522	(-5.259, 0.216)	-1.246	(-3.186, 0.693)
Baccalaureate	-0.575	(-2.707, 1.557)	-2.049	(-4.700, 0.603)	-1.474	(-3.280, 0.332)
University	-1.247	(-3.226, 0.731)	-3.904**	(-6.434, -1.374)	-2.657**	(-4.419, -0.893)
*Employment*						
Employed	Ref.	-	Ref.	-	Ref.	-
Unemployed	-0.347	(-1.360, 0.667)	0.123	(-1.536, 1.782)	0.470	(-0.911, 1.851)
Inactive	0.367	(-0.526, 1.259)	-1.157	(-3.006, 0.692)	-1.524	(-3.179, 0.131)
Cannabis use						
Nº of years	0.052	(-0.019, 0.123)	0.197**	(0.061, 0.333)	0.145*	(0.026, 0.264)
Smoked mix tobacco	1.019	(-0.091, 2.218)	13.815	(-1994, 2022)	12.796	(-1995, 2021)
Used at home	0.822	(-0.239, 1.883)	0.829	(-1.536, 3.193)	0.006	(-2.196, 2.209)
Used outdoors	0.633	(-0.114, 1.380)	1.371*	(0.058, 2.685)	0.739	(-0.376, 1.853)
Used in a vehicle	0.049	(-0.744, 0.842)	0.792	(-0.498, 2.083)	0.743	(-0.323, 1.809)
Reasons for use						
Expansion	-0.017	(-0.358, 0.322)	-0.464	(-1.068, 0.140)	-0.447	(-0.963, 0.070)
Coping	0.364	(-0.022, 0.751))	0.069	(-0.606, 0.744)	-0.295	(-0.874, 0.283)
Sources of supply						
Obtained for free	-1.018**	(-1.734, -0.301)	-1.965*	(-3.656, -0.275)	-0.947	(-2.521, 0.626)
Bought in a club	1.360***	(0.539, 2.181)	3.066***	(1.644, 4.487)	1.706**	(0.509, 2.903)
Home grown	1.526	(-0.131, 3.183)	0.513	(-2.046, 3.072)	-1.013	(-3.014, 0.998)
Adverse outcomes						
Psychological	0.472	(-0.372, 1.315)	-0.039	(-1.490, 1.412)	-0.511	(-1.748, 0.727)
Problems with family	-0.806	(-1.846, 0.235)	0.250	(-1.419, 1.919)	1.056	(-0.308, 2.420)
Financial	-0.348	(-1.139, 0.443)	-0.401	(-1.771, 0.968)	-0.053	(-1.209, 1.102)
Fines	0.454	(-0.286, 1.194)	0.612	(-0.704, 1.927))	0.157	(-0.979, 1.294)
CAST	0.095	(-0.002, 0.193)	0.318***	(0.147, 0.489)	0.223**	(0.077, 0.369)
Use of other substances						
Tobacco	0.113	(-0.631, 0.859)	0.993	(-0.617, 2.606)	0.879	(-0.579, 2.338)
Alcohol	-0.105	(-1.081, 0.870)	-1.434	(-2.971, 0.102)	-1.328*	(-2.587, -0.070)
Inhalants	1.491	(-0.105, 3.089)	2.021	(-0.218, 4.260)	0.530	(-1.159, 2.219)
Cocaine	-0.017	(-0.808, 0.774)	0.346	(-1.006, 1.698)	0.363	(-0.792, 1.517)
Amphetamine	0.471	(-0.481, 1.423)	0.937	(-0.679, 2.553)	0.466	(-0.907, 1.840)
LSD	1.162	(-0.295, 2.621)	1.782	(-0.568, 4.133)	0.619	(-1.303, 2.542)
Synthetic cannabin	0.558	(-1.724, 2.839)	1.235	(-1.774, 4.225)	0.678	(-1.496, 2.852)

*p < 0.05, **p < 0.01, *** *p* < 0.001. Sample size is 373

Compared to the moderately heavy group, the heavy group comprised more individuals in the 21–30 years age cohort (*b* = 1.413, *p* < 0.05). It was less probable that they obtained cannabis for free (*b*=-1.018, *p* < 0.01), and more likely that they acquired cannabis in a club (*b* = 1.360, *p* < 0.001). Compared to the moderately heavy group, those in the very heavy group showed a lower proportion of university graduates (*b*=-3.904, *p* = 0.01), more years of cannabis use (*b* = 0.197, *p* < 0.01), and used it outdoors more (*b* = 1.371, *p* = 0.05). It was less probable that they obtained cannabis for free (*b* = -1.965, *p* < 0.05), and more probable that they had bought it in a club (*b* = 3.066, *p* < 0.001), and had higher CAST scores (*b* = 0.318, *p* < 0.001). Compared to the heavy group, subjects in the very heavy group exhibited a lower proportion of university graduates (*b*=-2.657, *p* < 0.01) and more years of cannabis use (*b* = 0.145, *p* < 0.05). It was more probable that they purchased cannabis in a club (*b* = 1.706, *p* < 0.01) and had higher CAST scores (*b* = 0.223, *p* < 0.01). It was less probable that they had drunk alcohol in the past month (*b*=-1.328, *p* < 0.05). In summary, age, education, number of years of use, using outdoors, obtaining for free, buying cannabis in a club, CAST scores, and alcohol use were associated with cannabis use intensity.

## Discussion

The present study identified three latent classes of PWUC frequently characterized by consumption intensity. Age, education, years of use, and buying cannabis in a club emerged as correlates of class membership. No significant intergroup differences were observed regarding other factors—gender, unemployment, mode of administration, motives, and other substance use (except alcohol). The prevalence of CUD and dependence increased through the classes.

At least two previous LCA-based studies have shown that PWUC are a varied population, with DND users comprising three main groups or categories. Pearson et al. [24] conducted LCA on data collected from a sample of college students who had used cannabis in the past 30 days. They identified four latent classes using three variables related to the intensity of consumption and one more about cannabis-related problems. The largest class consisted of PWUC infrequently; the other three revealed increasingly frequent use and more negative consequences. They concluded that people using cannabis a few times monthly were distinct from DND users. Manning et al. [25] discovered five latent classes among a sample of 374 cannabis-using adults. The three variables used for the LCA were cannabis use frequency, quantity, and problems experienced. Three classes reported more heavy use patterns associated with increased adverse outcomes. Our study confirms that heavy users can be classified into three groups according to cannabis exposure. Besides, it shows that cannabis-related problems increase among DND users in parallel with use intensity. As such, use frequency may not be the key category for distinguishing heavy users. The number of daily doses (joints in our research) and whether users consumed cannabis throughout the waking day resulted in clear, distinct patterns of use and consequences. The heaviest users may be intoxicated throughout the waking day. However, for some DND users, especially if they consume lower doses at specific times, intoxication may not interfere much with their daily life.

In our study, the heaviest users were older, had used cannabis use for more years, and were less educated. Older users may have had more time to develop more heavy use patterns since there were no significant intergroup differences in terms of the age of cannabis use onset. Former research has interpreted early-onset and prolonged cannabis use as predictors of poorer educational outcomes and unemployment [39–42]. We only observed intergroup differences regarding education. Future studies should examine correlates between the three groups and other demographics such as gender or employment in larger samples of heavy users. For instance, in our research, 79% of the heaviest users were male, but the intergroup gender differences were not significant.

Many studies have reported associations between intensity (frequency and/or quantity) of cannabis use and cannabis-related problems [10, 24, 25, 43–45]. Our participants did not widely acknowledge cannabis-related problems. A third reported psychological and financial difficulties, and only 10% health damage. In contrast, the average CAST score was high (M = 10, SD = 4.4), and notable intergroup differences emerged. The proportion of individuals with scores ≥ 9 (indicating CUD) in the very heavy group was more than two times higher than in the moderately heavy group. This difference increased to more than three times when considering scores ≥ 12 (denoting dependence). These findings are consistent with former research, indicating that frequency of consumption is the most significant predictor of CUD, even when controlling for different products and modes of use [22, 46]. Additionally, nearly one in three PWUC frequently develops dependence [47]. Although using multiple products and modes of administration is not common in Spain, some users are under the effects of cannabis most of the day. Consumption intensity is a complex category that must reflect the number of hours under the influence of the substance.

Consistent with previous research [24, 25, 48, 49], enhancement, coping, expansion, and social motives were the most prevalent cannabis use reasons across classes. We did not observe significant intergroup differences regarding reasons for use in the multinomial logistic regression. These findings are consistent with those of Pearson et al. [24].

Concurrent use of other substances, except opioids, was common in our sample. Tobacco and alcohol use in the past month was prevalent across classes, and more than half reported having used at least one illicit drug other than cannabis in the past year (most commonly cocaine, followed by ecstasy and amphetamines). The prevalence of alcohol use in the past month was significantly lower in the very heavy group. Other studies have observed this polydrug use in daily users [50]. According to the literature, tobacco and cannabis seem to be complementary [51]. It is less clear if alcohol is a substitute for cannabis [52]. More research is needed to clarify the relationships between cannabis and other substances.

Spanish cannabis clubs may have become a principal supply source for the heaviest users in Spain. We found significant and broad intergroup differences regarding accessing cannabis clubs to obtain and use cannabis on their premises. Two previous studies of Spanish cannabis clubs [53, 54] found 77% and 68% of their members were daily users, respectively (samples N = 458 and N = 155). Most members were long-term cannabis users, and they did not change their use pattern after joining the club.

Some authors have pointed out that cannabis clubs could play a relevant role in implementing harm reduction practices [55, 56]. The preference of the heaviest users for this source of supply might support this proposal. However, in general, cannabis clubs have to fill some crucial gaps to implement a harm reduction policy: providing information on risks and harms, offering health support services for members, performing lab tests on the cannabis they supply, etc. [57]. Additionally, cannabis clubs must reconsider the maximum quantity of cannabis distributed monthly to each member—currently between 60 and 90 g [56]—, conduct follow-ups with frequent users, advise them to reduce their doses and frequency of use, and help problematic users access treatment and health advisory services. Based on our findings, clinical treatment interventions should also pay special attention to the use patterns of PWUC heavily since they are diverse and related to CUD. Further research will need to identify more correlates of class membership, which will enable more specific interventions for each heavy user class.

## Limitations

The study has several limitations. Firstly, we cannot know the representativeness of the sample. Network sampling is widely used to reduce biases in gathering samples of hard-to-reach and hidden populations, such as the one targeted in this study [58]. This study was not intended to provide prevalence estimates of different user groups that might exist among PWUC but rather to characterize DND cannabis users and identify correlates of class membership. Secondly, all data were self-reported measures, which must be considered when interpreting the results. Although self-report is an accepted method for obtaining population behavior information, individual bias and memory issues can compromise data accuracy [59]. Nonetheless, we have confidence in the validity of our main findings, which are consistent with former studies. We believe the set of questions assessing the quantity of cannabis used is reliable. We have more reservations about the results related to use of other substances. Future research will benefit from combining interview assessments, biological controls of cannabis use, and behavioral tasks to assess more accurate constructs. Thirdly, we could not measure THC content and other cannabinoids in the cannabis herb and resin used by participants, which are paramount to assessing the intensity of consumption and its consequences. However, a previous study [29] did not find large average differences in the potencies of these products in Spain. Nonetheless, future research will need to identify the potency of cannabis products to have better control of the study variables.

## Conclusion

This study suggests that PWUC heavily form three well-differentiated classes. Class membership was related to outcomes associated with cannabis use, including increased CUD and dependence. These findings are coherent with former research and highlight the necessity of considering the differences among heavy users to implement harm reduction policies, particularly in cannabis clubs, and clinical treatment of CUD.

## Data Availability

The datasets used and/or analyzed during the current study are available from the corresponding author on reasonable request.

## References

[1] UNODC (United Nations Office on Drugs and Crime). World Drug Report 2021. [Internet]. (United Nations publication, Sales No. E.21.XI.8); 2021. Available from: www.unodc.org/unodc/en/data-and-analysis/wdr2021.html

[2] EMCDDA (European Monitoring Centre for Drugs and Drug Addiction). European drug report 2021. Trends and developments [Internet]. Luxembourg: Publications Office of the European Union. ; 2021. Available from: https://www.emcdda.europa.eu/system/files/publications/13838/TDAT21001ENN.pdf

[3] OEDA (Observatorio Español de las Drogas y las Adicciones). Informe 2021. Alcohol, tabaco y drogas ilegales en España [Report 2021. Alcohol, tobacco and illegal drugs in Spain] [Internet]. Madrid: Ministerio de Sanidad. Delegación del Gobierno para el Plan Nacional sobre Drogas; 2021. Available from: https://pnsd.sanidad.gob.es/profesionales/sistemasInformacion/sistemaInformacion/pdf/2019-20_Informe_EDADES.pdf

[4] Callaghan RC, Sanches M, Benny C, Stockwell T, Sherk A, Kish SJ. Who consumes most of the cannabis in Canada? Profiles of cannabis consumption by quantity. Drug Alcohol Depend [Internet]. 2019;205:107587. Available from: 10.1016/j.drugalcdep.2019.107587 10.1016/j.drugalcdep.2019.107587 31600617

[5] Caulkins JP, Pardo B, Kilmer B. Intensity of cannabis use: Findings from three online surveys. Int J Drug Policy [Internet]. 2020;79:102740. Available from: 10.1016/j.drugpo.2020.102740 10.1016/j.drugpo.2020.102740 32334336

[6] Chan GCK, Hall W (2020). Estimation of the proportion of population cannabis consumption in Australia that is accounted for by daily users using Monte Carlo Simulation. Addiction.

[7] Burns RM, Caulkins JP, Everingham SS, Kilmer B (2013). Statistics on cannabis users skew perceptions of cannabis use. Front Psychiatry.

[8] Midgette G, Davenport S, Caulkins JP, Kilmer B. What America’s users spend on illegal drugs, 2006–2016 [Internet]. Santa Monica, California: RAND Corporation; 2019. Available from: https://www.rand.org/content/dam/rand/pubs/research_reports/RR3100/RR3140/RAND_RR3140.pdf

[9] Van Laar M, Frijins T, Trautmann F, Lombi L, Cannabis. market: User types, availability and consumption estimates. In: Trautmann F, Kilmer B, Thurnbull P, editors. Further insights into aspects of the illicit EU drugs market [Internet]. Luxembourg: Publications Office of the European Union; 2013. p. 73–182. Available from: http://ec.europa.eu/justice/anti-drugs/files/eu_market_full.pdf

[10] Zeisser C, Thompson K, Stockwell T, Duff C, Chow C, Vallance K (2012). A ‘standard joint’? The role of quantity in predicting cannabis-related problems. Addict Res Theory.

[11] Curran HV, Freeman TP, Mokrysz C, Lewis DA, Morgan C, Parsons JA (2016). Keep off the grass? Cannabis, cognition and addiction. Nat Rev Neurosci.

[12] Hall WD (2015). What has research over the past two decades revealed about the adverse health effects of recreational cannabis use?. Addiction.

[13] NASEM (National Academies of Sciences, Engineering and Medicine). The health effects of cannabis and cannabinoids: The current state of evidence and recommendations for research [Internet]. Washington, D.C.: The National Academies Press. ; 2017. Available from: https://www.nap.edu/catalog/24625 28182367

[14] Volkow ND, Swanson JM, Evins AE, DeLisi LE, Meier MH, Gonzalez R (2016). Effects of cannabis use on human behavior, including cognition, motivation, and psychosis: a review. JAMA Psychiatry.

[15] WHO (World Health Organization). The health and social effects of nonmedical cannabis use [Internet]. 2016. Available from: http://who.int/substance_abuse/publications/msbcannabis.pdf?ua=1

[16] Lorenzetti V, Hindocha C, Petrilli K, Griffiths P, Brown J, Castillo-Carniglia Á (2022). The International Cannabis Toolkit (iCannToolkit): a multidisciplinary expert consensus on minimum standards for measuring cannabis use. Addiction.

[17] Gamella JF, Jiménez Rodrigo ML (2003). El consumo prolongado de cannabis. Pautas, tendencias y consecuencias [Long-term cannabis use: patterns, trends, and consequences].

[18] Van der Pol P, Liebregts N, Brunt T, Van Amsterdam J, De Graaf R, Korf D (2014). Cross-sectional and prospective relation of cannabis potency, dosing and smoking behaviour with cannabis dependence: an ecological study. Addiction.

[19] Tomko RL, Baker NL, McClure EA, Sonne SC, McRae-Clark AL, Sherman BJ (2018). Incremental validity of estimated cannabis grams as a predictor of problems and cannabinoid biomarkers: evidence from a clinical trial. Drug Alcohol Depend.

[20] Craft S, Winstock A, Ferris J, Mackie C, Lynskey MT, Freeman TP (2020). Characterising heterogeneity in the use of different cannabis products: latent class analysis with 55 000 people who use cannabis and associations with severity of cannabis dependence. Psychol Med.

[21] Davis CN, Slutske WS, Martin NG, Agrawal A, Lynskey MT. Identifying subtypes of cannabis users based on simultaneous polysubstance use. Drug Alcohol Depend [Internet]. 2019;205(April):107696. Available from: 10.1016/j.drugalcdep.2019.107696 10.1016/j.drugalcdep.2019.107696 PMC6893147 31726429

[22] Gunn RL, Aston ER, Sokolovsky AW, White HR, Jackson KM. Complex cannabis use patterns: Associations with cannabis consequences and cannabis use disorder symptomatology. Addict Behav [Internet]. 2020;105:106329. Available from: 10.1016/j.addbeh.2020.106329 10.1016/j.addbeh.2020.106329 PMC7104573 32044680

[23] Krauss MJ, Rajbhandari B, Sowles SJ, Spitznagel EL, Cavazos-Rehg P (2017). A latent class analysis of poly-marijuana use among young adults. Addict Behav.

[24] Pearson MR, Bravo AJ, Conner BT, Anthenien AM, Bravo AJ, Conner BT (2017). Distinguishing subpopulations of marijuana users with latent profile analysis. Drug Alcohol Depend.

[25] Manning K, Garey L, Paulus DJ, Buckner JD, Hogan JBD, Schmidt NB (2019). Typology of cannabis use among adults: a latent class approach to risk and protective factors. Addict Behav.

[26] Fischer B, Rehm J, Irving H, Ialomiteanu A, Fallu J-S, Patra J (2010). Typologies of cannabis users and associated characteristics relevant for public health: a latent class analysis of data from a nationally representative canadian adult survey. Int J Methods Psychiatr Res.

[27] Van der Pol P, Liebregts N, De Graaf R, Korf D, Van den Brink W, Van Laar M (2011). The dutch Cannabis Dependence (CanDep) study on the course of frequent cannabis use and dependence: objectives, methods and sample characteristics. Int J Methods Psychiatr Res.

[28] Van der Pol P, Liebregts N, De Graaf R, Korf DJ, Van den Brink W, Van Laar M (2013). Validation of self-reported cannabis dose and potency: an ecological study. Addiction.

[29] Casajuana Kögel C, Balcells-Olivero MM, López-Pelayo H, Miquel L, Teixidó L, Colom J (2017). The standard joint unit. Drug Alcohol Depend.

[30] Matali Costa J, Simons J, Pardo M, Lleras M, Pérez A, Andión O (2018). Spanish version validation of the Marihuana Motives measure in a drug-consuming adolescent sample. Adicciones.

[31] Simons J, Correia CJ, Carey KB, Borsari BE (1998). Validating a five-factor marijuana motives measure: relations with use, problems, and alcohol motives. J Couns Psychol.

[32] Benschop A, Liebregts N, Van der Pol P, Schaap R, Buisman R, Van Laar M (2015). Reliability and validity of the Marijuana Motives measure among young adult frequent cannabis users and associations with cannabis dependence. Addict Behav.

[33] Legleye S, Karila L, Beck F, Reynaud M (2007). Validation of the CAST, a general population Cannabis abuse screening test. J Subst Use.

[34] Legleye S, Guignard R, Richard J, Ludwig K, Pabst A, Beck F (2015). Properties of the Cannabis abuse screening test (CAST) in the general population. Int J Methods Psychiatr Res.

[35] DGPNSD (Delegación del Gobierno para el Plan Nacional sobre Drogas). Consumo problemático de cannabis en estudiantes españoles de 14–18 años: validación de escalas [Problematic cannabis use in Spanish students aged 14–18: validation of scales] [Internet]. 2009. Available from: http://www.pnsd.msssi.gob.es/profesionales/publicaciones/catalogo/catalogoPNSD/publicaciones/pdf/ConsProblematico_cannabis.pdf

[36] Cuenca-Royo AM, Sánchez-Niubó A, Forero CG, Torrens M, Suelves JM, Domingo-Salvany A (2012). Psychometric properties of the CAST and SDS scales in young adult cannabis users. Addict Behav.

[37] Weller BE, Bowen NK, Faubert SJ (2020). Latent class analysis: a guide to best practice. J Black Psychol.

[38] Masyn KE, Little TD (2013). Latent class analysis and finite mixture modeling. The Oxford handbook of quantitative methods in psychology.

[39] Boden JM, Dhakal B, Foulds JA, Horwood LJ (2020). Life-course trajectories of cannabis use: a latent class analysis of a New Zealand birth cohort. Addiction.

[40] Thompson K, Leadbeater B, Ames M, Merrin GJ (2019). Associations between marijuana use trajectories and educational and occupational success in young adulthood. Prev Sci.

[41] Zhang C, Brook JS, Leukefeld CG, Brook DW (2016). Trajectories of marijuana use from adolescence to adulthood as predictors of unemployment status in the early forties. Am J Addict.

[42] Beverly HK, Castro Y, Opara I (2019). Age of first marijuana use and its impact on education attainment and employment status. J Drug Issues.

[43] Walden N, Earleywine M (2008). How high: quantity as a predictor of cannabis-related problems. Harm Reduct J.

[44] Norberg MM, Mackenzie J, Copeland J (2012). Quantifying cannabis use with the Timeline Followback approach: a psychometric evaluation. Drug Alcohol Depend.

[45] Foster KT, Arterberry BJ, Iacono WG, Mcgue M, Hicks BM (2018). Psychosocial functioning among regular cannabis users with and without cannabis use disorder. Psychol Med.

[46] Swan C, Ferro MA, Thompson K. Does how you use matter? The link between mode of use and cannabis-related risk. Addict Behav [Internet]. 2021;112:106620. Available from: 10.1016/j.addbeh.2020.106620 10.1016/j.addbeh.2020.106620 32911353

[47] Van der Pol P, Liebregts N, De Graaf R, Korf DJ, Van den Brink W, Van Laar M (2013). Predicting the transition from frequent cannabis use to cannabis dependence: a three-year prospective study. Drug Alcohol Depend.

[48] Casajuana Kögel C, López-Pelayo H, Oliveras C, Colom J, Gual A, Balcells-Oliveró MM (2021). The relationship between motivations for cannabis consumption and problematic use. Adicciones.

[49] Buckner JD, Zvolensky MJ, Crosby RD, Wonderlich SA, Ecker AH, Richter A (2015). Antecedents and consequences of cannabis use among racially diverse cannabis users: an analysis from ecological momentary assessment. Drug Alcohol Depend.

[50] Hughes JR, Fingar JR, Budney AJ, Naud S, Helzer JE, Callas PW (2014). Marijuana use and intoxication among daily users: an intensive longitudinal study. Addict Behav.

[51] Agrawal A, Budney AJ, Lynskey MT (2012). The co-occurring use and misuse of cannabis and tobacco: a review. Addiction.

[52] Risso C, Boniface S, Subbaraman MS, Englund A (2020). Does cannabis complement or substitute alcohol consumption? A systematic review of human and animal studies. J Psychopharmacol.

[53] Parés-Franquero Ò, Jubert-Cortiella X, Olivares-Gálvez S, Díaz-Castellano A, Jiménez-Garrido DF, Bouso JC (2019). Use and habits of the protagonists of the story: Cannabis Social Clubs in Barcelona. J Drug Issues.

[54] Arnoso A, Elgorriaga E. Estudio de las pautas de consumo de cannabis en los clubes sociales de cannabis y evaluación de su eficacia (2016) [Study of cannabis use patterns in cannabis social clubs and evaluation of their efficacy (2016)] [Internet]. San Sebastián: Fundación Renovatio; 2016. Available from: https://docs.wixstatic.com/ugd/1bbd46_860b4d80f0a24ac39e20dde40404a79d.pdf

[55] Belackova V, Tomkova A, Zabransky T (2016). Qualitative research in spanish Cannabis Social Clubs: “The moment you enter the door, you are minimising the risks. Int J Drug Policy.

[56] Decorte T, Pardal M, Queirolo R, Boidi MF, Sánchez Avilés C, Parés Franquero Ò (2017). Regulating Cannabis Social Clubs: a comparative analysis of legal and self-regulatory practices in Spain, Belgium and Uruguay. Int J Drug Policy.

[57] Obradors-Pineda A, Bouso JC, Parés-Franquero Ò, Romaní JO. Harm reduction and cannabis social clubs: Exploring their true potential. Int J Drug Policy [Internet]. 2021;97:103358. Available from: 10.1016/j.drugpo.2021.103358 10.1016/j.drugpo.2021.103358 34252786

[58] Bell DC, Erbaugh EB, Serrano T, Dayton-Shotts CA, Montoya ID (2017). A comparison of network sampling designs for a hidden population of drug users: Random walk vs. respondent-driven sampling. Soc Sci Res.

[59] Rendon A, Livingston M, Suzuki S, Hill W, Walters S (2017). What’s the agreement between self-reported and biochemical verification of drug use? A look at permanent supportive housing residents. Addict Behav.

